# “New” Antigenic Targets and Methodological Approaches for Refining Laboratory Diagnosis of Antiphospholipid Syndrome

**DOI:** 10.1155/2015/858542

**Published:** 2015-03-19

**Authors:** Roberta Misasi, Antonella Capozzi, Agostina Longo, Serena Recalchi, Emanuela Lococo, Cristiano Alessandri, Fabrizio Conti, Guido Valesini, Maurizio Sorice

**Affiliations:** ^1^Dipartimento di Medicina Sperimentale, Sapienza Università di Roma, Viale Regina Elena 324, 00161 Roma, Italy; ^2^Dipartimento di Medicina Interna e Specialità Mediche, Reumatologia, Sapienza Università di Roma, Viale del Policlinico 155, 00161 Roma, Italy

## Abstract

Antiphospholipid antibodies (aPLs) are a heterogeneous group of antibodies directed against phospholipids or protein/phospholipid complexes. Currently, aPLs are assessed using either “solid-phase” assays that identify anticardiolipin antibodies and anti-*β*2-glycoprotein I antibodies or “liquid-phase” assay that identifies lupus anticoagulant. However, in the last few years, “new” antigenic targets and methodological approaches have been employed for refining laboratory diagnosis of antiphospholipid syndrome (APS). In this review the potential diagnostic value of antibodies to domains of *β*2-GPI, prothrombin/phosphatidylserine, vimentin/cardiolipin, protein S, protein C, annexin A2, annexin A5, and phospholipid antigens is discussed. Moreover, new technical approaches, including chemiluminescence, multiline dot assay, and thin layer chromatography (TLC) immunostaining, which utilize different supports for detection of aPL, have been developed. A special focus has been dedicated on “seronegative” APS, that is, those patients with a clinical profile suggestive of APS (thromboses, recurrent miscarriages, or foetal loss), who are persistently negative for the routinely used aPL. Recent findings suggest that, in sera from patients with SN-APS, antibodies may be detected using “new” antigenic targets (mainly vimentin/cardiolipin) or methodological approaches different from traditional techniques (TLC immunostaining). Thus, APS represents a mosaic, in which antibodies against different antigenic targets may be detected thanks to the continuously evolving new technologies.

## 1. Introduction

The antiphospholipid syndrome (APS) was first described in 1980s [1, 2] and the term was first coined to describe patients with recurrent thrombosis or pregnancy complications. Although APS was first reported in systemic lupus erythematosus (SLE), later on it became obvious that SLE was not a necessary condition for its occurrence [3].

The diagnosis of the disease has witnessed a remarkable evolution over the course of the past 25 years and it has been shown that antibodies to phospholipids are the main responsible agents of the disease, hence its name.

In 1999, international experts developed consensus criteria on the clinical and laboratory criteria for “definite APS” that became known as the Sapporo Criteria. These criteria were subsequently updated in 2006 at a meeting in Sydney and are now referred to as updated Sapporo or Sydney Criteria. The clinical criteria include objectively confirmed venous, arterial or small vessel thrombosis, or pregnancy complications that may be attributed to placental insufficiency, including pregnancy loss or premature birth. The laboratory criteria require that a positive laboratory test for antiphospholipid antibodies (aPLs) is found on 2 or more occasions at least 12 weeks apart. The aPLs recognized in the international criteria include anticardiolipin (aCL) antibody (IgG or IgM) exceeding 40 IgG or IgM phospholipid units or anti-*β*2-glycoprotein I (*β*2-GPI) antibodies (IgG or IgM) at titers exceeding the 99th percentile and lupus anticoagulant (LA) detected according to guidelines published by International Society on Thrombosis and Haemostasis (ISTH) [4, 5].

However, aPLs are a heterogeneous group of antibodies directed against phospholipids or protein/phospholipid complexes. Currently, aPLs are assessed within the laboratory, using either “solid-phase” assays that identify anticardiolipin (aCL) antibodies and anti-*β*2-glycoprotein I antibodies or “liquid-phase” assay that identify LA [6].

The results of ELISA procedure to detect aCL and anti-*β*2-GPI are typically reported either in arbitrary units or as GPL or MPL units, which, respectively, reflect the level of reactive immunoglobulins (Ig) class G or M based on a polyclonal or monoclonal antibody calibrator standards. The lack of agreed standards leads to extraordinarily high interlaboratory or intermethod variability. This high intermethod variability, at least in part, helps to explain the relative low clinical utility of aCL assay when assessed on a global basis and in terms of association with adverse clinical effects such as thrombosis. One more reason for the aCL assay to be of such relatively low clinical utility is that the assay is “oversensitive” to aPL, detecting both clinically and nonclinically relevant form of aPL, as well as “low-titer” aPL that may not be associated with adverse clinical features of APS, that is, during infections [7].

Therefore, the currently available assays for the detection of aPL, while maintaining a diagnostic validity, lack prognostic value and they are unsuitable as indicators for preventive therapies because all three assays do not always detect the desired population of antibodies, but they measure a mixture of clinically relevant and irrelevant antibodies. One of the challenges in the serological testing of APS is not only to design assay but also to develop assays that assess the risk of thrombosis recurrence [7].

More recently, a global APS score (GAPSS) was developed [8]; the authors propose an attempt of scoring the aPL profile. Higher accuracy of the aPL score is obtained when all aPL tests are included. However, even though all of the tests are not accessible, a partial aPL score would help clinicians in the risk stratification and in making decisions about therapeutic approaches.

Moreover, the American Heart Association recently issued guidelines for the evaluation of novel biomarkers for cardiovascular diseases. The guidelines propose standards for the critical appraisal of novel risk markers that are developed for clinical use. It has been proposed [8] that these guidelines could be applied to APS preexisting markers in order to assess whether the current panel of diagnostic tests complies with them. To provide a risk stratified approach, the assays should meet the following: standardization criteria of aPL tests and the aPL assays should correlate with clinical symptoms and should have a predictive risk value for future thrombotic events or pregnancy complications and add predictive information to already established risk markers.

Nowadays, the currently available aPL assays do not completely fulfill these simple criteria, and so it is, therefore, appropriate to continue the research in order to improve biotechnology available at the case and to introduce novel assays able to overcome these methodological shortcomings. However, many efforts are being made in the direction of risk stratification, starting from the studies on the genesis of a*β*2-GPI antibodies, through the different approaches to measure them in the clinical laboratory, in order to provide additional and relevant serologic information to properly assess the risk of thrombosis.

## 2. Different Antibody Specificities in APS

As reported above, aPLs are a heterogeneous group of autoantibodies directed against negatively charged molecules and a combination of phospholipids and/or protein-complexed phospholipids, including not only *β*2-GPI [6, 9, 10] but also different anionic phospholipids, proteins, or phospholipid-protein complexes, such as prothrombin/phosphatidylserine [11], vimentin/cardiolipin [12], protein S [13], protein C [14], annexin A5 [15], annexin A2 [16], oxidized low-density lipoproteins (LDL), lysobisphosphatidic acid (LBPA), and sulfatides [17–19].

### 2.1. Antibodies to *β*2-GPI

#### 2.1.1. “Classical” Anti-*β*2-GPI

Since 1990, McNeil et al. identified that the binding of aPL to cardiolipin requires the presence of a protein cofactor, the apolipoprotein *β*2-GPI, which is a member of the complement control family and is considered as a natural inhibitor of coagulation [20].

*β*2-GPI, which circulates as a monomer, consists of five highly homologous protein repeats or “sushi domain.” The fifth sushi domain contains a phospholipid insertion loop, which enables it to interact with anionic phospholipids, such as phosphatidylserine or cardiolipin. Furthermore, *β*2-GPI can exist in two conformations in plasma, closed circular form and open form [9]. The circular conformation is maintained by interaction between the first and fifth domain of *β*2-GPI; in the open conformation a cryptic epitope in the first domain becomes exposed, enabling antibody binding. Antibody-*β*2-GPI complexes bind to a variety of receptors (e.g., Toll-like receptors 2 and 4, annexin A2, glycoprotein 1b*α*, and LRP8 in the LDL receptors) on different cell types, including endothelial cells, platelets, monocytes, and trophoblasts [21]. This binding may trigger intracellular signalling and inflammatory responses [22, 23].

*β*2-GPI has natural anticoagulant properties* per se*, but it may also bind to oxidized low-density lipoprotein (oxLDL) to neutralize its proinflammatory effects. oxLDL/*β*2-GPI complexes are immunogenic and it has been demonstrated that autoantibodies against oxLDL/*β*2-GPI complexes strongly correlate with arterial thrombosis in patients with SLE and APS [24].

Anti-*β*2-GPI antibodies represent a highly specific test for diagnosis of APS. Indeed, IgG anti-*β*2-GPI antibodies were reported to be more specific than IgG aCL for the APS [25]. However, positive anti-*β*2-GPI results have been detected in SLE (11–39%) and infectious diseases. On the contrary, IgG anti-*β*2-GPI antibodies appear to be less sensitive than IgG aCL; interestingly 3–10% of patients sera are only anti-*β*2-GPI antibody positive. Contradictory results have been reported on the prognostic value of anti-*β*2-GPI [26–28]. Correlation between anti-*β*2-GPI positivity alone and thrombosis or fetal loss is controversial, probably due to the poor standardization of this assay. It has been shown that the quality of purified *β*2-GPI used and the coating of the ELISA plate strongly influence the result of the assay. Indeed, coating with *β*2-GPI to ELISA plates will result in the masking of certain epitopes on *β*2-GPI that might be important for the recognition by antibodies. The use of different types of ELISA plates, hydrophobic or hydrophilic, will result in different adsorption of *β*2-GPI to the plate and exposure of a different set of epitopes. In addition, Koike and coworkers [29, 30] demonstrated that the orientation of adsorbed *β*2GPI on the plastic surface of an ELISA plate may affect antibody binding.

It is, therefore, evident that autoantibodies against *β*2-GPI are a heterogeneous population of antibodies containing subclasses directed against every domain of *β*2-GPI.

#### 2.1.2. Antibodies to Domains of *β*2-GPI

Anti-*β*2-GPI antibodies comprise a family of antibodies which recognize different epitopes of the protein. In the last few years, several data showed that antibodies to domain I (DI), specifically glycine40 and arginine43, showed a good correlation with thrombosis and pregnancy morbidity [31]. However, other studies reported that the epitope may comprise a larger region on domains I and II. Anti-*β*2-GPI antibodies with DI specificity were found in the majority of APS patients and were significantly associated with LA and venous thrombosis [32]. In addition, a subsequent multicenter study also revealed an association with the obstetric manifestations of the syndrome [33], suggesting that anti-D1 antibodies may represent a more predictive profile [34]. The fact that anti-DI antibodies are directed against the immunodominant epitope of *β*2-GPI is also supported by pathogenic studies in animal models. Infusion of synthetic peptide DI was shown to protect naïve mice from the thrombogenic effects of aPL IgG [35]. Moreover, anti-DI MoAbs IgG was shown to induce clotting and fetal loss in naïve mice, providing the first evidence of the pathogenic role of anti-DI antibodies [36]. This demonstration was supported by the observation that anti-DI MoAb induced clotting via complement activation, only after administration of lipopolysaccharide. Additional epidemiological studies support the diagnostic value of anti-DI antibodies as compared to antibodies directed against different *β*2-GPI domains. In fact, anti-DI antibodies may cluster in patients with autoimmune diseases [37], whereas aPL-positive asymptomatic subjects, individuals with leprosy, or children with atopic dermatitis have been shown to preferentially recognize epitopes on DIV or DV [38]. Thus, it was suggested that ratio between anti-*β*2-GPI-DI and anti-*β*2-GPI DIV/DV IgG can be able to discriminate between anti-*β*2-GPI linked to an autoimmune profile and antibodies occurring in other conditions.

Anti-DI antibodies were detected in a large proportion of APS patients, resulting in a sensitivity of 85% and a specificity of 99.5% [38]. Therefore, although further studies are needed, it seems clear that we are on track to draw definite conclusions on the diagnostic and prognostic value of anti-DI antibodies.

#### 2.1.3. IgA Anti-*β*2-GPI

Several studies recently analysed the role of the IgA isotype. In particular, the pathogenicity of IgA aPL was demonstrated in animals models. Mice, injected with IgA aPL from patients with APS, developed thrombosis [39].

Moreover, numerous studies have also investigated possible associations between raised levels of anti-*β*2-GPI and clinical manifestations of APS. In particular, the presence of anti-*β*2-GPI and of IgA aCL appeared to be related to thrombosis and thrombocytopenia; women with unexplained recurrent spontaneous abortions and fetal death were shown to express IgA anti-*β*2-GPI, in the absence of LA [40]. In a recent multicentre cohort study, which analysed 588 sera from SLE patients, 75 sera were found exclusively positive for this isotype [41], showing a good correlation with clinical manifestation(s) of APS. Recently, a study on 156 patients fulfilling clinical criteria for APS (independently of serological markers) showed that 22.4% of them were positive for IgA anti-*β*2-GPI alone [42]. At the end, IgA anti-*β*2-GPI antibodies are also considered an independent marker for development of several atherosclerotic manifestations, such as acute myocardial infarction and acute cerebral ischemia [43].

Thus, according to these observations, it is agreed that patients with SLE and/or APS should be also tested for IgA anti-*β*2-GPI antibody, especially when the other tests are persistently negative.

### 2.2. Antibodies to Protein S/Protein C

Since 1993, Oosting et al. suggested the existence of subpopulations of aPL, which are directed to complexes of phospholipids and different plasma proteins involved in the coagulation system [44]. Thus, they proposed a thrombogenic mechanism for aPL in which antibodies bind to the complexes of PL and coagulation proteins, including prothrombin, protein S, and protein C. Several studies reported the presence of antiprotein S and/or protein C in APS patients [13, 14], though with less sensitivity and specificity as compared to IgG aCL. In addition, levels of these antibodies (IgM and IgG) appeared to be related to pregnancy disorders and preeclampsia [45]. However, conflicting results were obtained concerning their clinical significance.

More information is available on their pathogenic role. We demonstrated that anti-protein S antibodies are able to inhibit PS activity [46]. Anti-protein C antibodies were shown to be associated with resistance to endogenous protein C activation and a severe thrombotic phenotype in APS [14]. Thus, it was hypothesized that anti-protein C antibodies may represent a different subset from those that define APS. Indeed, these antibodies do not cosegregate with the presence of APS-defining aPL. However, high-avidity anti-protein C antibodies were present only in patients with APS, with 80% being classified as category I. It suggests that high-avidity anti-protein C antibodies might play a role as an adjunctive risk factor in APS patients with a severe thrombotic phenotype.

### 2.3. Antibodies to Prothrombin and Prothrombin/Phosphatidylserine Complex

Prothrombin is a plasma glycoprotein which converted to thrombin by extrinsic thromboplastin during the second stage of blood clotting. Since 1995, anti-prothrombin antibodies were reported in patients with APS syndrome (50–90%) and this antigen was considered as a potential cofactor protein for aPL detection. However, conflicting results were obtained concerning their clinical significance. It may be due to the use of different ELISA kits for detection of anti-prothrombin antibodies. Some investigators used irradiated plates and buffers containing detergents, while others used non-*γ*-irradiated plates in addition to the different blocking solutions. Finally, in recent years, two different prospective studies validated the role of anti-prothrombin antibodies in predicting the risk of thrombosis in patients with APS [47, 48]. In particular, a long-term longitudinal study (15 years) identified IgG anti-prothrombin antibodies as the most useful predictor of thrombosis in SLE patients [48]. Further studies demonstrated that antiprothrombin is also capable of binding to prothrombin/phosphatidylserine complex. Again, conflicting findings on their clinical significance were found. Recently, Bertolaccini et al. conclusively revealed a positive association between the presence of antiprothrombin/phosphatidylserine Ab (IgG and/or IgM) and arterial or venous thrombosis [11, 49]. Moreover, antiprothrombin/phosphatidylserine Ab were shown to have higher specificity and sensitivity than conventional aCL, showing a highly significant correlation with LA. Thus, antiprothrombin, mainly antiprothrombin/phosphatidylserine Ab, may be considered a useful tool and can be potentially used as both confirmatory diagnostic markers and indicators of the risk of thrombosis. In addition, they may be useful to identify some patients with clinical features suggestive of APS but persistently negative to all the classical tests for detection of aPL [50].

### 2.4. Antibodies to Vimentin/Cardiolipin Complex

In search for potential antigenic targets for diagnosis of APS, a proteomic approach identified vimentin as the main endothelial molecule recognized by aPL. Vimentin is a cytoskeleton intermediate filament protein ubiquitously expressed. Surface-expressed forms of vimentin have recently been localized on the surface of apoptotic neutrophils and T cells [51], activated macrophages [52], platelets [53], vascular endothelial cells [54], and others. The mechanism by which vimentin reaches the cell surface, in which domains are exposed, and its function at the surface remain unknown. However, vimentin and cardiolipin can interact at the surface of apoptotic cells to form an immunogenic particle [55]. Vimentin-cardiolipin binding may be attributable to electrostatic interaction between positive charged amino acids of vimentin and negative charged of cardiolipin.

Based on these data, APS sera were tested for the presence of anti-vimentin/CL antibodies [12]. Results showed persistent presence of IgG and/or IgM anti-vimentin/CL in 92.5% and 80% of patients with APS. However, although the presence of these antibodies may be considered quite sensitive in these patients, it is not highly specific, since they were also detected in a proportion of patients with SLE or RA. However, detection of these antibodies may represent a useful tool mainly in those patients with clinical features suggestive of APS in which the classical tests for detection of aPL are persistently negative.

### 2.5. Antibodies to Annexin A5 and Annexin A2

Annexins are a family of ubiquitous calcium-dependent PL-binding proteins. Annexin A5 (AnnA5) is an anticoagulant protein mainly found in trophoblasts and vascular endothelial cells. *β*2-GPI- dependent aPL may interfere with the protective binding of AnnA5 to the endothelium, thus leading to thrombosis [15]. A novel coagulation assay to determine AnnA5 resistance has been developed: several independent studies revealed that a large proportion of APS patients were AnnA5-resistant [41]. Interestingly, resistance to AnnA5 anticoagulant activity inversely correlates with titres of IgG antibodies targeting DI in both thrombotic and obstetric manifestations of SLE [41].

Anti-AnnA5 antibodies have been proposed to be associated with the clinical features of APS, including thrombosis and recurrent miscarriages. However, the clinical correlation of anti-AnnA5 with pregnancy-related morbidity is still controversial, due to inconsistent results among studies.

Annexin A2 (AnnA2) is an important binding site for *β*2-GPI on the surface of endothelial and monocytes cells. Different studies demonstrated that cross-linking or clustering of A2-bound *β*2-GPI leads to cellular activation with consequent expression of procoagulant phenotype and inflammatory cytokines [22, 56]. In fact, AnnA2 was showed to be a component of a multimolecular signaling complex on endothelial cell surface [57].

Antibodies directed to AnnA2 were detected in APS patients with an occurrence significantly higher as compared to healthy individuals or patients with SLE without thrombosis. However, sensitivity of this test is quite low, since recent data suggest that about 25% of APS patients are positive [58]; anti-AnnA2 antibodies were also detected in patients suffering from other autoimmune conditions, thus lowering their specificity [16, 59]. Further studies are required to determine their clinical significance and diagnostic value to discriminate clinical subgroups of APS patients.

### 2.6. Antibodies to Phospholipid Antigens

Several authors revisited the diagnostic and analytical properties of antibodies directed against phospholipids antigens, including IgG and IgM antibodies directed against phosphatidic acid (PA), phosphatidylinositol (PI), phosphatidylserine (PS), phosphatidylethanolamine (PE), cardiolipin (CL), lyso-bis-phosphatidic acid (LBPA), and sulfatides. The diagnostic and prognostic role of several autoantibodies targeting negatively charged PLs other than CL have been evaluated [6, 41, 60]. In particular, three anionic phospholipids, such as PS, PI, and PA, found in the most cell membranes, are well characterized as antigens in the APS. In this regard, however, testing for these aPL antibodies does not improve the probability of diagnosing APS compared with classical criteria tests. Nevertheless, some investigators have suggested that testing for aPS, aPI, and aPA antibodies may help to identify women with recurrent pregnancy loss [61–64]. However, it has been reported that mainly aPS have a particular relevance in obstetric APS. In fact, several results, obtained using* in vitro* model systems, showed that antibodies directed against PS inhibit trophoblast development and invasion. Moreover, aPS may retard syncytiotrophoblast formation and decrease the hCG synthesis [65]. Indeed, several studies have shown that the use of these three antibody groups in APS diagnosis remains controversial. In fact, it has been reported that aCL broadly cross-reacts with both aPS and aPI [66–68].

Antibodies directed against PE have been described, in some instances, as the sole aPL, in patients with APS clinical manifestations and for this reason they deserve particular attention [69, 70]. PE is a zwitterionic PL, mainly located in the inner leaflet of biological membranes; some types of aPE antibodies may bind to high molecular weight kininogen, leading to the formation of antibody-PE-kininogen trimolecular complex that enhances thrombin-induced platelet aggregation. Different results demonstrate the interest in aPE investigation with regard to obstetrical complications. In fact, aPE have been reported to be significantly more frequent in women with unexplained early fetal loss than in either those with explained early fetal loss or healthy mothers. Moreover, in several studies relationship between aPE antibodies and other clinical features of APS has been reported and the prevalence of these antibodies in patients with unexplained venous thromboses was demonstrated [71, 72].

Several reports have described in APS patients the presence of antibodies to an unconventional phospholipid, such as LBPA. This is a lipid restricted to the late endosomes, and it was reported that it is recognized within these internal membranes, by aPL antibodies and this binding to intracellular LBPA may constitute a possible mechanism for the thrombogenic effects. In particular, aLBPA may explain their direct pathogenic role in APS syndrome, by altering endosomal sorting and vesicular trafficking [17, 73]. Interestingly, aLBPA are present in the sera of a large number of APS patients, showing similar sensitivity and specificity compared to anti-*β*2-GPI antibodies and close association with LA [18, 74, 75]. In this regard, it was demonstrated that aLBPA are internalized, react with late endosomes containing LBPA, and induce an intracellular redistribution of *β*2-GPI [76]. Moreover, aLBPA were shown to be able to exert LA activity* in vitro*. However, results obtained assessing aLBPA antibodies in APS patients do not provide yet a real advantage for diagnosis and/or identification of different clinical and laboratory subsets. In fact, in the clinical practice these antibodies, despite being specific for the syndrome, always show a lower sensibility compared to other tests, including aCL and anti-*β*2-GPI antibodies [77].

Another specificity identified in APS sera is represented by antibodies to sulfatides. These molecules, acid glycosphingolipids, are physiologically involved in the hemostatic process. Sulfatides are present in various tissues and cells and they are one of the major families of lipids in the serum. *β*2-GPI is able to bind sulfatides and antibodies directed against sulfatide/*β*2-GPI complex may also react with CL/*β*2-GPI complex. These autoantibodies may contribute not only to the hemostatic abnormalities but also to other clinical features of APS, such as neurological symptoms and abortions, since sulfatides are present in nerve tissue and in female genital tract [19].

Taken together, all these observations indicate that, concerning heterogeneity of “antiphospholipid antibodies,” the term aPL is actually incorrect, because the most clinically important antibodies are not directed against pure PL, but they bind PL in association with their protein cofactors [78–80].

## 3. Different Technical Approaches for APS Diagnosis

New technical approaches, which utilize different supports for detection of aPL, have been developed in the last few years (Table 1). They might be useful to refine our knowledge on the antigen specificities of “antiphospholipid antibodies,” since in all these systems the antigenic presentation of proteins and/or phospholipid/protein complexes are quite different as compared to standard ELISA.

### 3.1. Chemiluminescence Assay

Automated Chemiluminescence Immunoassay (CLIA) is an alternative method to enzyme linked immunosorbent assay (ELISA). Automatization can improve the reproducibility and reduce interlaboratory variation [81]. In this regard, chemiluminescence that is fully automated represents several advantages over semimanual ELISA techniques for its implementation in a routine laboratory. Analysers that provide a chemiluminescent technology use a two-step immunoassay method and the specific antibodies, present in the sample, bind to the solid-phase represented by magnetic particles coated with the antigen. When the reagents that trigger the chemiluminescent reaction were added, emitted light is measured by the optical system as relative light units (RLUs). This signal is directly proportional to the aPL antibodies concentration in the sample [82, 83].

Different authors evaluated the comparison of automated systems applying chemiluminescent technology to aPL antibodies, mutually and with ELISA, with the aim of investigating the equivalence in their diagnostic performance. Therefore, these fully automated and computerized analysers significantly reduce the hands-on time compared to the labour-intensive ELISA assays. Moreover, the diagnostic relevance of the new assays was evaluated by verifying their ability to correctly classify patients with definite APS [83, 84]. aCL antibodies have limitations according to robustness, reproducibility, standardization, and clinical relevance; on the other hand, anti-*β*2-GPI and LA tests are highly specific but not very sensitive [81]. Thus, CLIA, though less sensitive than ELISA, appears to be more useful for identifying APS patients. This difference was not very surprising, since these automated systems differ from ELISA for the antigenic presentation of proteins and phospholipid/protein complex (mainly CL/*β*2-GPI) on magnetic particles compared to the surface of microtitre wells. Moreover, binding of the *β*2-GPI protein on the solid-phase is crucial and determines antigen density and orientation/conformational change of the protein [85]. Thus, the new different coating system is a key element mostly for aCL antibodies detection and could explain, together with the amplification reaction of the chemiluminescent principle, the extremely high titres for aPL antibodies detected with the automated analysers. Therefore, these new chemiluminescent assay panels show good performance characteristics [86], showing a sensitivity of 100% and specificity of 72.3% for patients with APS [87]. Thus, it may represent a useful tool to detect mostly relevant IgG aCL according to revised Sydney criteria.

### 3.2. Multiline Dot Assay

Multiline dot assays (MLDA) or other bead-based multiplex techniques candidate as alternatives to asses several aPL antibodies simultaneously, employing different solid-phases for binding of antigens. In fact, phospholipids or protein cofactors were sprayed onto polyvinylidene difluoride (PVDF) membranes in lines for immobilization. Processed strips were then read out and results can be assessed semiquantitatively by densitometric evaluation. However, in this technique, the use of hydrophobic membranes as a solid-surface appears to offer a distinct solid-phase reaction environment for the assessment of aPL antibodies [88–90]. Interestingly, these novel solid phases should provide the same properties of ELISA supports regarding conformational changes of immobilized cofactors. In fact, immunodot assay has been employed for the assessment of disease-specific anti-*β*2-GPI antibodies, revealing no difference with ELISA data [91]. Conversely, anionic phospholipids immobilized on such membrane appear to generate a different reaction environment for the aPL antibodies binding. In fact, in contrast to the solid-phase in ELISA, the porous structure of the hydrophobic membrane may hide the large hydrophobic part of phospholipids, and this can lead to a denser presentation of the hydrophilic part of phospholipids on the membrane surface which interacts with cofactors and specific autoantibodies. Moreover, it may be assumed that the membrane immobilization mimics the* in vivo* presentation on anionic phospholipids and that the formation of multiple interconnected immune complexes on an appropriate lipid surface might be important for a strong, amplified, and bivalent aPL antibodies binding [92].

Thus, MLDA for aPL antibodies profiling is an effective multiparameter test system for the simultaneous semiquantitative detection of several autoantibodies in one sample and appears to candidate as a potential solution for the cost-effectiveness of aPL tests, as reported for multiples assessment of autoantibodies in other autoimmune diseases like SLE and rheumatoid arthritis [93, 94].

Results obtained by various authors, as regards this assay, were in good agreement with ELISA data, without no statistical difference on the laboratory diagnosis of APS. Moreover, IgM antibodies to PL, detected by MLDA, demonstrated a significant association with cerebrovascular symptoms. Thus, this technique is readly available, single-step sensitive diagnostic tool and is recommended to identify patients at higher risk, although standardization of assay remains a challenge [95, 96].

### 3.3. TLC Immunostaining

Thin layer chromatography (TLC) is a nonquantitative technique which has been employed for detection of aPL antibodies. This method has been firstly used in 1994 and includes three main steps: the antigen separation, immunostaining with patients' sera, and, finally, detection of immunoreactivity [97]. For the first step, phospholipids run on aluminium-backed silica gel performance thin layer chromatography (HPLC) plates, using an appropriate eluent system, then chromatograms are incubated with sera, and finally immunoreactivity is assessed by chemiluminescence reaction. Thus, this is an easy and suitable laboratory approach, capable of revealing simultaneously reactivity of autoantibodies, from patients' sera, directed against various purified PL molecules that show a different antigenic exposure as compared to ELISA [98]. This technique seems to be less sensitive but more specific than ELISA in both autoimmune and infectious diseases. For this purpose, TLC immunostaining exploits the fact that antigens run on aluminium-backed silica gel plates mimicking the exposure of phospholipid to binding proteins [99, 100]. Thus, this is a further technical approach able to provide a useful tool for clarifying the immunological specificity of aPL [50, 101].

## 4. “Seronegative” APS

There is a close relationship between autoimmunity and autoantibodies, even though some patients with autoimmune diseases might be persistently negative for disease-specific autoantibodies. These conditions have been defined as “seronegative” autoimmune diseases (e.g., seronegative rheumatoid arthritis). “Seronegative” autoimmune diseases may represent a practical problem because they are often difficult cases [102, 103].

As reported above, diagnosis of APS requires the combination of at least one clinical and one laboratory criterion. Nevertheless, in daily clinical practice it is possible to find patients with a clinical profile suggestive of APS (thromboses, recurrent miscarriages or foetal loss, and some noncriteria features), who are persistently negative for the routinely used aCL, anti-*β*2-GPI, and LA. For these cases the term “seronegative APS” (SN-APS) has been proposed [104, 105]. Several possible explanations for such “seronegative” cases have been suggested: either the diagnosis is wrong, previously positive aPL tests have become negative, or, as seems most likely, the current range of tests is inadequate. The latter may depend on limits of the traditional technical approaches or on the existence of different antigenic targets.

As deep vein thrombosis, myocardial infarction, and stroke are major causes of morbidity and mortality in APS due to the high risk of recurrence, it is mandatory to identify among the so-called SN-APS patients those who need long-term secondary thromboprophylaxis. Likewise, since APS is now recognised as the commonest treatable cause of recurrent miscarriage, for women with a history of recurrent early abortions or fetal loss, a diagnosis of APS addresses them towards treatments which significantly improve the rate of live births.

### 4.1. Antibody Specificities in “Seronegative” APS

Therefore, new antigenic targets or methodological approaches to detect aPL in SN-APS have been investigated. In particular, anti-prothrombin antibodies have been reported as the sole antibodies detected in few patients who had SLE and a history of thrombosis but were persistently negative for aCL or LA [106].

Recently, with a proteomic approach, analyzing endothelial cell-surface membrane proteins, vimentin/cardiolipin complex was identified as a “new” target antigen of SN-APS [12]. Serum IgG anti-vimentin/cardiolipin antibodies, detected by ELISA, were found not only in a large proportion of SN-APS patients (55%) but also in almost all APS patients. The test performed with a second sample obtained at least 12 weeks from the previous one confirmed the same result in all SN-APS patients.

A different laboratory technique, capable of detecting aPL by immunostaining on TLC plates, has been proposed. TLC immunostaining relies upon the different partition characteristics of phospholipids between the surface (stationary phase) and mobile solvent phase for different solvent polarities. In this case, the binding of phospholipid to solid-phase mainly involves both electrostatic and hydrophobic interactions. Thus, this nonquantitative technique is able to identify the reactivity of serum aPL with a different antigenic exposure compared to ELISA methods [97].

TLC immunostaining was recently used for detection of aPL (CL, antilysobisphosphatidic acid, and antiphosphatidylethanolamine antibodies) in a group of 36 patients with a clinical picture suggestive of APS, that is, vascular thrombosis and/or pregnancy morbidity associated with several noncriteria APS features (e.g., livedo reticularis, thrombocytopenia, cognitive dysfunctions, migraine, and seizures), persistently negative for the routinely used aPL [101]. The presence of aPL was identified in about 60% of SN-APS patients. Interestingly, a strong correlation was observed between the three aPL specificities demonstrated by TLC immunostaining. In order to verify the possible pathogenic role of the autoantibodies, it was demonstrated that purified IgG from sera of SN-APS patients induced IRAK serine phosphorylation with consequent NF-*κ*B activation [101].

More recently, sera from 24 SN-APS patients were analysed for aPL by TLC immunostaining, for anti-vimentin/cardiolipin antibodies by ELISA, and for anti-annA5 and anti-prothrombin antibodies by ELISA and dot blot, with the aim of identifying the best screening combination to detect aPL in SN-APS patients [50]. In this cohort of SN-APS patients, the results obtained by TLC immunostaining showed the presence of aCL in 54.2% of cases. In addition, 45.8% of them showed serum antibodies (IgG class) against vimentin/cardiolipin, 12.5% against prothrombin, and 4.2% against annA5. Taken together, these findings showed that in 19 out of 24 SN-APS (79.2%) at least one aPL/cofactor antibody was detected using the assays under test. The combination of two of the tested methodological approaches, TLC immunostaining for aCL and ELISA for anti-vimentin/cardiolipin complex antibodies, was able to detect aPL/cofactors in about two-thirds of SN-APS patients with thrombosis or pregnancy morbidity, with a small additional gain when also performing ELISA for prothrombin and annA5. Moreover, recent evidence showed that anti-DI, IgA aCL, or IgA anti-*β*2-GPI may also pick up a small proportion of patients with SN-APS [107]. In conclusion, the combined use of different tests can be very useful for identification of autoantibodies in these patients, starting with TLC immunostaining and antivimentin/cardiolipin ELISA and eventually anticofactors ELISA to reveal the hidden reactivity of the conventional aPL detection. However, although these approaches improve our diagnostic possibilities, we are still unable to detect autoantibodies in a percentage of SN-APS patients. Thus, since other unidentified cofactors may be involved in sera reactivity, further studies could shed light on “new” antigenic specificities in SN-APS.

## 5. Conclusion

APS remains a significant diagnostic challenge for clinicians across a wide range of specialities, largely due to issues related to laboratory testing as well as the expanding range of reported clinical manifestations of APS.

Although it is the clinician looking after the patient that ultimately makes the diagnosis, laboratory plays a major role at many stages of the process. The laboratory issues include limitations in detailed knowledge by both clinical and laboratory staff regarding the “complete” range of available aPL tests, as well as ongoing problems with assay reproducibility and standardization [6].

Anti-phospholipid antibodies play a major pathogenic role inducing clinical manifestations; however, there is growing evidence that inflammatory stimuli are pivotal for triggering thrombosis, while tissue distribution of antigenic target, as well as posttranscriptional modifications or the fine specificity of anti-*β*2-GPI, may influence the type of clinical events or even their occurrence [108]. Several issues with regard to the mechanisms that precipitate the various clinical manifestations of APS are still debated; however, we may say that the composite of aPL findings actually provides the greatest risk stratification tool for associated adverse events such as thrombosis [109]. Thus, although LA is a stronger predictor of risk than either aCL or anti-*β*2-GPI, the greatest risk appears to be when patients have multiple positivities (LA, aCL, anti-*β*2-GPI), it was found that triple positivity was associated with thrombosis in 87% of cases while in the other profiles the association was around 50% [110–112].Recently, it has been reported [113] that patients with triple positivity for aCL, LA, and anti-*β*2-GPI had a greater risk of thrombotic events than those who were positive for only one or two of these specificities. In any case, a combination of aPL tests should be considered when discussing the risk of thrombosis/pregnancy morbidity [114]. Several attempts have been made in order to identify the individual risk of thrombosis in patients positive for aPL [8, 115, 116].

In addition, “noncriteria” aPL tests (included in Table 2) have been proposed to be relevant in APS [41]. Moreover, autoantibody presence or absence might subclassify APS according to the association of these antibodies with clinical manifestations [10]. Further studies are in progress in order to validate “noncriteria” aPL as risk factors for clinical events.

Moreover, a diagnosis of SN-APS has been suggested for those patients presenting with clinical manifestations characteristic of APS, but with persistently negative aPL tests, including anti-*β*2-GPI and LA. By employing a different methodological approach for detection of aPL, such as TLC immunostaining, as well as new antigenic targets of APS, such as vimentin/cardiolipin complex, it may be possible to detect aPL/cofactors in about two-thirds of SN-APS patients with thrombosis or pregnancy morbidity [50].

Nevertheless, much remains to be done. For example, we need to train clinicians better in terms of test requests to avoid inappropriate testing that is costly and which may lead to false-positive diagnosis of APS and adverse patient treatments. But mostly we need to develop better assays and to improve standardization for APS diagnosis.

**Figure 1:**
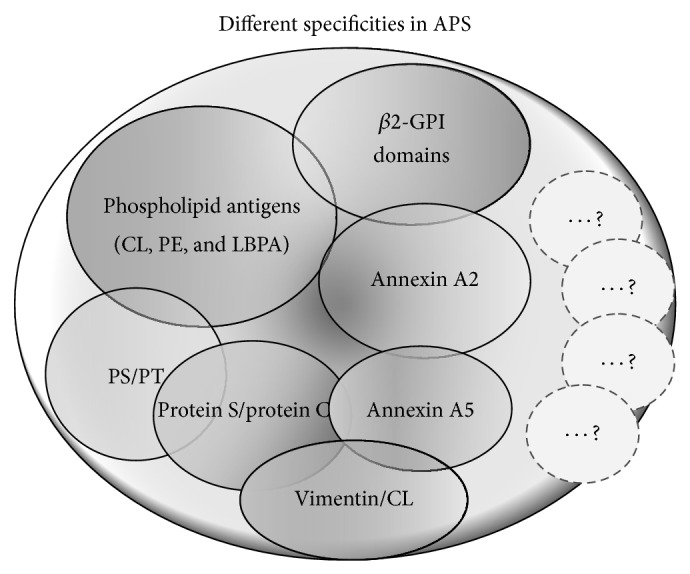


We can conclude that APS is still an evolving field and that, ultimately, a more holistic approach to the diagnosis of APS is needed. New technologies may represent useful tools for diagnosis of immunological disorders, contributing to a better efficiency and accuracy in the diagnosis of autoimmune diseases. The new diagnostic approaches should point out the risk stratification of the disease, taking into account first the potential combinations/panels of available aPL tests, including the issues of different entities of “seropositive” APS, “seronegative” APS, and non-APS aPL-positivity. This can be achieved also thanks to the various technologies that allow us to identify “new” epitopes, revealed through the typology of antigen presentation (Figure 1). Moreover, it will allow us to review new treatment strategies for APS that may target different pathways of coagulation and immunomodulation.

## Figures and Tables

**Table 1:** Different technical approaches for aPL detection.

Assays	Technical characteristics	Main antibodies detected	References
ELISA	Protein and/or PL/protein complex coated on surface of polystyrene plates microtitre wells	Anti-CL/*β*2-GPI (IgG, IgM, IgA) Anti-PE Anti-*β*2-GPI (IgG, IgM, IgA) Anti-*β*2-GPI-DI Anti-vimentin/CL Anti-PT/PS Anti-AnnA5 Anti-AnnA2	[6, 9, 10] [67–69] [39, 40, 42, 43] [31–36] [51–55] [11, 47–50] [41] [16, 22, 41, 56–59]

Chemiluminescence	Antigenic presentation of proteins and/or PL/protein complex on magnetic particles Binding on this solid phase determines peculiar density, orientation, and conformational changes of antigenic epitopes	Anti-CL/*β*2-GPI Anti-*β*2-GPI	[81–87]

Dot blot	Phospholipid or proteins immobilized onto PVDF membranes: these hydrophobic membranes offer a distinct reaction environment, hiding hydrophobic phospholipid groups and exposing hydrophilic part	Anti-PL Anti-protein cofactors	[88–91]

TLC immunostaining	Antigens run on aluminum-backed silica gel plates mimicking the exposure of phospholipid to binding proteins Chromatograms are incubated with sera and finally immunoreactivity is assessed by chemiluminescence reaction	Anti-PL	[50, 98–101]

aPL: anti-phospholipid antibodies; anti-CL: anti-cardiolipin antibodies; anti-*β*2-GPI: anti-beta2-glycoprotein I antibodies; anti-*β*2-GPI-DI: anti-beta2-glycoprotein I antibodies domain I antibodies; anti-PT/PS: anti-prothrombin/phosphatidylserine antibodies; anti-vimentin/CL: anti-vimentin/cardiolipin antibodies; anti-AnnA5: anti-annexin A5 antibodies; anti-AnnA2: anti-annexin A2 antibodies; anti-PE: anti-phosphatidylethanolamine antibodies; PVDF: polyvinylidene difluoride membranes; TLC: thin layer chromatography.

**Table 2:** Different antibody specificities in APS.

Autoantibody	Clinical associations	Prevalence	References
Anti-CL/*β*2-GPI	IgG isotype related to thrombotic events and mainly associate with cerebrovascular accidents and myocardial infarction; IgG titers correlate with brain thrombosis	80–90%	[10, 117]

Anti-*β*2-GPI	May inhibit protein C and annexin V anticoagulant activity and boost platelets adhesion and may play a role in development of thromboembolic phenomena	60–90%	[10, 118]

Anti-*β*2-GPI-DI	Associate with thrombosis and obstetric manifestations (more than antibodies directed to other domains)	27–85%	[31–33, 38, 107]

IgA anti-*β*2-GPI	May be related to miscarriages, thrombocytopenia, livedo reticularis, pulmonary hypertension, and seizure; present in association with SLE	20–25%	[40–42, 107, 119]

Anti-protein S/protein C	Associate with a severe thrombotic phenotype	14%/49%	[14]

Anti-PT/PS	Strongly associate with thrombosis and pregnancy complications	50–90%	[11, 49, 63]

Anti-vimentin/CL	May be related to recurrent thrombosis and pregnancy morbidity	88–92.5%	[12, 50]

Anti-AnnA5	Clinical correlation with pregnancy-related morbidity is still controversial	30.4%	[41, 120]

Anti-AnnA2	Alter profibrinolytic activity and correlate with thrombotic events	25.0%	[58]

Anti-PE	May affect protein C pathway and bind coagulation factor XI, may correlate with thrombotic events, and may be present in patients with unexplained thrombosis or early fetal loss	73–95%	[69–72]

Anti-LBPA	Associate with aCL, anti-*β*2-GPI, and LA and do not provide yet a real advantage for diagnosis and/or identification of different clinical subsets	67%	[73–76]

aPL: anti-phospholipid antibodies; anti-CL: anti-cardiolipin antibodies; anti-*β*2-GPI: anti-beta2-glycoprotein I antibodies; anti-*β*2-GPI-DI: anti-beta2-glycoprotein I antibodies domain I antibodies; anti-PT/PS: anti-prothrombin/phosphatidylserine antibodies; anti-vimentin/CL: anti-vimentin/cardiolipin antibodies; anti-AnnA5: anti-annexin A5 antibodies; anti-AnnA2: anti-annexin A2 antibodies; anti-PE: anti-phosphatidylethanolamine antibodies, anti-LBPA: anti-lyso-bis-phosphatidic acid antibodies; SLE: systemic lupus erythematosus.
